# A description of data sets to determine the innovative diversification capacity of farm households

**DOI:** 10.1016/j.dib.2016.07.007

**Published:** 2016-07-09

**Authors:** Terence Mc Fadden

**Affiliations:** University College Dublin, Ireland

**Keywords:** Innovation, Diversification, Farm, Household, Policy, Environment

## Abstract

This data represents research activities carried out in Co. Offaly and Co. Mayo, Ireland, to identify farm household innovative diversification behavior and policy/institutional actor capacity roles in support. The data sets are overlain with household and agency data from the two study areas to describe levels of innovative diversification capacity by individual socio-economic farm household profile. The data sets summarize the public policy discussions on rural innovation and diversification and policy actor response requirements, and incorporate both qualitative and quantitative data set combinations. The data are used to assess policy/institutional actors’ roles and farm households’ capacity for innovation at the farm household/institution interface in support of sustainable rural business innovations on-farm and diversification.

## **Specifications Table**

**Table:** 

Subject area	Farm Household Innovative Capacity, Innovational behavior
More specific subject area	Policy strategy, business activity
Type of data	Tables, figure
How data was acquired	Interviews, audio and visual recording, on-site repeat visits, written
Data format	Raw and analyzed
Experimental factors	Material and non-material evidence was recorded by repeated visits to farm households, by interview, farm walks, respondent/researcher interaction, observing and recording physical landscape features and behaviors, and reviewing household financial accounts. The observational/experiential research method from 2007–2016 was selected to identify innovation activity seasonally comparable annually.
Experimental features	The farm data are categorized by farm household numbers assigned to the farm households by postal survey (in [4]) entitled, ‘Farm Household Profile Data’. This data presents a profile of each farm household researched including their involvements with development agencies, to figure out the innovative capacity of different socio-economic household profiles in two of Ireland׳s most distinct counties – midland county Offaly and coastal county Mayo.
Data source location	Co. Offaly and Co. Mayo, Ireland
Data accessibility	Data are included in this paper

**Value of the data**

• The data show overall differences in innovation activity encountered in Co. Offaly and Co. Mayo comparatively, including the characteristics present in the households being profiled in relation to the households’ engagements with policy actors.
• The data derived from field work presents the methodology used in the interests of policy makers and researchers to evaluate innovative capacity in different institutional scales and policy jurisdictions applying these methods. The barriers to more widespread innovation were assessed using the methods at diverse geographical scales and institutional scales.
• The numerical and qualitative evidence of innovative and non-innovative categorization in each geography and by individual household, enabled comparisons at the micro and macro scales, and cross-disciplinary discussion on innovative capacity.
• The overall findings from both county jurisdictions are comparative. They are informing policy and rural development strategies for sustainable business promotion and the development supports necessary at international and European Union (EU) policy levels.
• The data was necessary to build up a profile of innovative, potential innovative, and non-innovative farm household diversifiers and determine the reasons for behavioral change over time.

## 1. Data

**Fig. 1:**
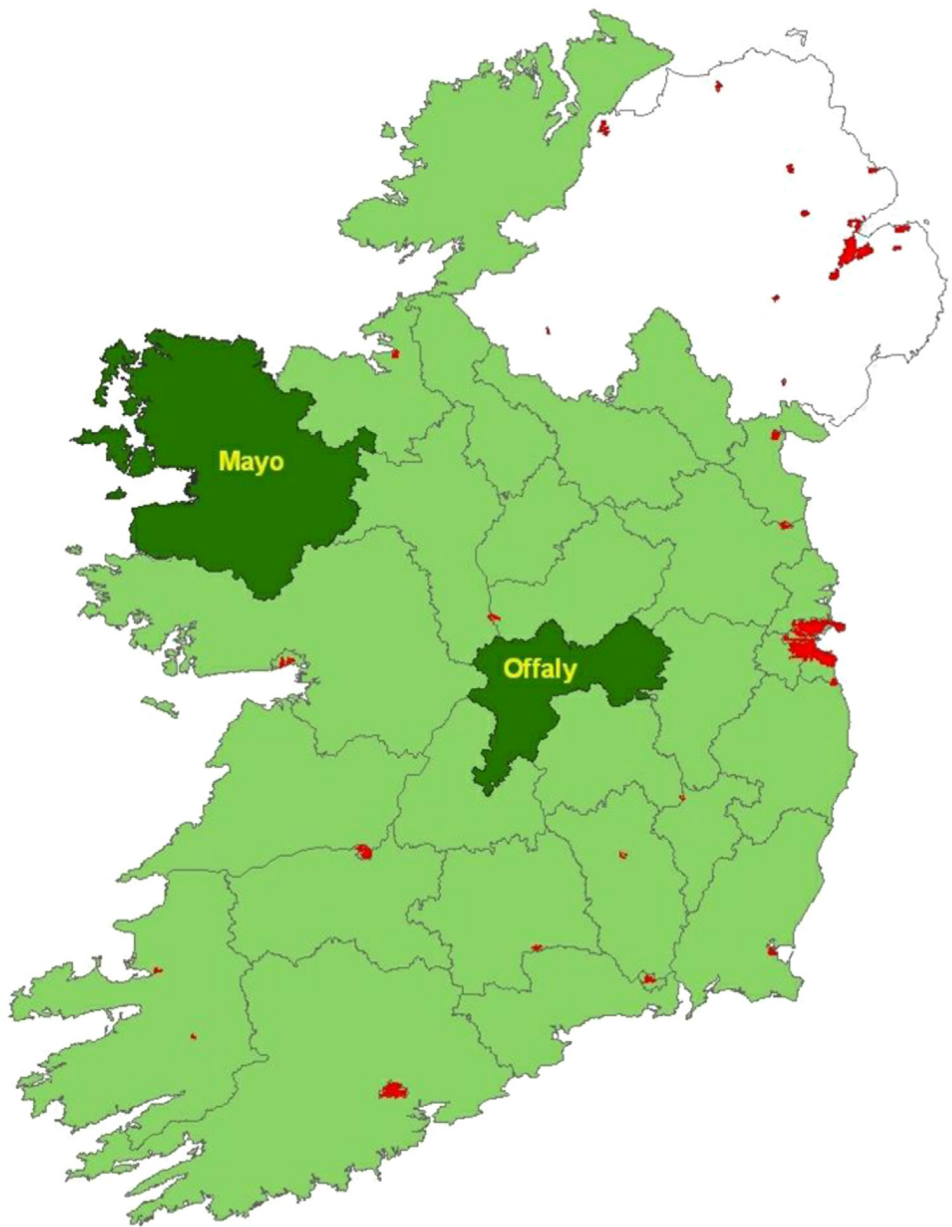
Research Site Map – Co. Offaly and Co. Mayo. Original source: Ordnance Survey Ireland.

Table 1 (in [4]) provides an outline of the categories applied and the grading system applied; Supplementary Table 1a presents the households’ profiled; Fig. 1 shows the research locations; Appendix A, Appendix A presents the agency/extension and analytic procedure leading from the main topics to the specific research topic; Appendix A, Appendix A ‘Category Index’ presents a Summary of the policy issues in the appraisal of decision processes in relation to farm households’ capacity for innovation; Appendix A, Appendix A shows the analytic procedure leading from the main topic of the research (see [4]) to the specific questions; Appendix A, Appendix A presents an overview of the farm households characteristics with emphasis on common characteristics.

## 2 Experimental design, materials and methods

### 2.1 Brief introduction of data source and processing methods

#### 2.1.1 Data resource

Three-hundred postal surveys were sent to farm households in Offaly and Mayo. Twenty-four postal surveys were sent to the same types of development agencies and extension services operating in both counties in support of farm innovation, diversification and business startup. The sample of the thirty-seven households (Appendix A, Appendix Aa) were filtered from the household postal survey data, and the agency interviews (Appendix A, Appendix A). The results of this filtering of data and defining the research categories are presented in the paper *‘Exploring the concept of farm household innovation capacity in relation to farm diversification in policy context’* [4]**.**

The data presented in this paper from farm households and development agencies/extension services in Co. Offaly and Co. Mayo, represent the observational/experiential methods applied (see also [4]). With a physical presence in the research field, the data methods identify and contextualize changes in farm household behavior, apparent stasis (non-business/innovation activity) in some households, and records activity information and physical data in decision making and the effects. The types of supporting business activity – networking, planning, business supports, households’ life cycle and objectives – are identified.

Fig. 1 illustrates the overall geographical territory in which the field research was undertaken. The research locations are the total areas of research by county, and location. ‘Fig. 1 Map of the study areas’ in [4] shows in more detail the research areas by main road network and main towns.

#### 2.1.2 Data processing

The quantitative research findings determined through the grid index method in Table 1 (in [4]) entitled ‘Household Capacity Index’, represent the thirty-seven households interviewed and graded. A score of 0–10 per the assigned characteristics identified as most important to innovation, are the result of repeat farm visits and interviews with farmers and policy actors (see [4]). The three distinct categories of farm household profiles are presented: innovative diversifier, non-innovative diversifier, and potential innovative diversifier. The evidence of business activity and related characteristics are linked numerically by corresponding household to Appendix A, Appendix Aa**.** *Farm Household Profile Data*, whereby each household profile is presented individually.

### 2.2 The presence of innovation activity in both geographies

Physical evidence of diversification activity was photographed, interviews were recorded and transcribed, and scores assigned by category on Excel. The objective was to assess the potential capacity of farm households to innovate. Essential innovative characteristics required this method of identification. The innovative and non-innovative characteristics common to each category correspond with the evidence from the physical/material farm environments, corresponding interviews, and ‘hard-copy’ evidence (account information). Focusing on individual households of different socio-economic/cultural status was necessary to determine the ‘intrinsic’ qualities which apparently make innovative business outcomes more likely, including technological, policy and institutional attributes supportive of entrepreneurial/innovative activities. Actor domains sympathetic to innovative outcomes formed essential research targets (see also [2]) to the research objectives. The total data were compared by household with the policy jurisdictions in which the households farmed. Supplementary Table 1a pinpoints also the presence (and non-presence) of innovative activity by household type by policy jurisdiction.

### 2.3 The extent of innovative diversification by household

The extent of innovative diversification by household represents data on the levels of education, networking activities and the characteristics of the households by profiling each household (a biography of each household is made) in relation to the evidence for business activity on-farm. The total capacity for innovation by county are also separately identifiable by county in relation to the policy environment׳s supportive roles [4]. The actual numbers of households identified and the percentage of involvement with policy actors are summarized and identifiable by county (Supplementary Table 1a).

### 2.4 The capacity for innovative activity by household

The data reveals the capacity for innovative activity by household in relation to the proportional levels of networking the households maintain and policy actor relationships the households maintain, the scores of which are in Table 1 (in [4]). The households’ total individual capacity for innovation within the categories are determined by their management strategies, business selection (suitability of the innovation/diversification/business to the particular household from the farm/household evidence), and by agency involvement (levels of supports planned or received, verified agency roles and inputs). The average levels of agency involvement with households are averaged by household, as are the management strategies and businesses selected by the households concentrating on households’ decision processes. Supplementary Table 4 – The farm-household interview schedule showing the analytic procedure from the main topic to specific questions – shows the data acquisition process which informed the household interviews and agency interviews. Supplementary Table 5 – Overview of characteristics of postal survey respondents and interview participants – presents an overview of the farm household data to reveal the characteristics of the different socio economic household profiles to include in abbreviated form the data on the different households and farm profiles and farm activity profiles.

### 2.5 The presence of innovative activity by household

To show the innovative and diverse capability of households (their capacity for innovation) within the geographic sample, (Table 1) *‘The Household Capacity Index’* (in [4]) was introduced to measure the essential strategies innovative households undertake and the degree to which innovative processes are representable qualitatively and quantitatively. The index varies between 0 and 100. When all 37 households were scored, the data was reviewed and the three categories could be clearly identified (Innovative Diversifiers; Potential Innovative Diversifiers; Non-Innovative Diversifiers) (Appendix A, Appendix Aa). The best examples of innovative diversifiers scored 72 and above out of a possible 100; the potential innovative diversifiers scored from 40 to 71, while those who were non-innovators scored below 39.

### 2.6 Use of the Methods

All the information and recommendations gathered during the pilot and subsequent field research phases were used to develop the interview guide (Supplementary Table 4). The sample was then selected according to “theoretical sampling”, a method derived from grounded theory based on analytic questions and comparisons, pinpointing ‘people and places’ to maximize the chances of discovering variations among concepts ([5]). The ‘Grounded Theory’ research approach was mainly concerned with research questions for which no direct information from previous research was available and therefore the initial (pilot study) research did not start with a specific theoretical hypothesis (see also [3], [1]). The facts of the appraisal of innovative diversification capacity awaited full exploration, because it emerged as a neglected research topic qualitatively which led to the researchers using this methodological approach in this research. A SWOT analytic approach was not considered sufficient based on the variations in business trajectories and development support processes.

A summary of the policy issues (Supplementary Table 3) found affecting the appraisal of decision processes in relation to farm households’ capacity for innovation provided the opportunity not only to use the most frequently repeated concepts in the empirical data, but also to have a qualitative/quantitative measurement capacity which addressed overlaps and discrepancies between policy positions. A summary of the agency/extension service interview structure, showing the analytic procedure leading from the main topics to the specific (in Supplementary Table 2), shows the connections made between categories and sub-categories which constitute the parameters of the more general issues interrogated. For, example, the potential for deficits in understanding and thus supporting a more innovative and diverse rural economy, absence of linkages between and within development agencies, the absence of policy oriented farm research focused on the farm household/institution interface, and the need for interdisciplinary research and performance capacity within an environment of change and uncertainty.

## Footnotes

Appendix A Supplementary data associated with this article can be found in the online version at doi:10.1016/j.dib.2016.07.007.
