# Supplementary material for: A description of data sets to determine the innovative diversification capacity of farm households
Source: Data Brief. 2016 Jul 9;8:1088–93. doi: 10.1016/j.dib.2016.07.007 (PMC4970492; doi:10.1016/j.dib.2016.07.007)
Supplement: Supplementary file 1 — Supplementary material [file mmc1.docx]

No Conflict of Interest
