# Supplementary material for: A description of data sets to determine the innovative diversification capacity of farm households
Source: Data Brief. 2016 Jul 9;8:1088–93. doi: 10.1016/j.dib.2016.07.007 (PMC4970492; doi:10.1016/j.dib.2016.07.007)
Supplement: Supplementary file 2 — Supplementary material [file mmc2.zip › Zip File JRS June 27/Table 1a - Farm Households by Category.docx]

**Table Ia. Farm Household Profile Data**

**(May = Mayo; Off = Offaly. (ID) = Innovative Diversification; (I) = Diversifier).**

| **Innovative Diversifiers = 11 Households** | | | | | | | | | | | | | | | | | | |  |
| --- | --- | --- | --- | --- | --- | --- | --- | --- | --- | --- | --- | --- | --- | --- | --- | --- | --- | --- | --- |
| **Household Profile.** | | | | | | **Land Area + Farming by Type – (ID) or (D).** | **Farm Activities – Number & Type** | | | **Social Networking: Extension Services/Agencies involved: Number Resources Exploited.** | | | **Sustainability/Development Strategy.** | | | **Grid Score: Innovation (out of 100)** | | |  |
| 1. | | | (H 43 May) 60 + years old Gender – Female Children - 2 (male & female); Age - 30 yrs +;  Education (Ed) – Third Level | | | 260 acres  Grazing & tillage | Seven  Museum, organic cattle & crops; conference centre; guest accommodation. | | | Extensive social networking. Teagasc; LEADER; Failte Ireland; Organics Group Member; Marketing of natural & cultural resources from off-farm locally & internationally | | | Long-term plan; Employment generation on-farm, & locally off-farm. | | | 95**%** | | |  |
| 2. | | | (H 52 Off) 50 + years old, male & female, three children (17 yrs +); Ed - Third & fourth level | | | 380 acres forestry grazing, tillage, cash crops | Nine equestrian (ID), accommodation (ID), cattle, sheep, farm shop (ID), stables (ID), walks (ID), treks (ID), organics | | | Extensive networking Teagasc, LEADER, Failte Ireland natural & cultural resources from on-farm locally | | | Long-term planning Employment generated locally, on and off-farm. | | | 95**%** | | |  |
| 3. | | | (H 53 Off) 45 years old;  Male dual partnership. ED – third level. | | | 280 acres  Grazing, cash crops | Three  Forestry, Organic Veg, Direct marketing. | | | Established business network, direct marketing, no extension services current, local markets, natural resources | | | Long-term planning, on-farm employment, direct product delivery to off farm markets. | | | **100%** | | |  |
| 4. | | | (H 11 Off) 30 yes + male and female; Two children under 17 yrs, Ed – Second level | | | 10 acres; Cash crops, organic box business | Two; organic veg, supplier of cardboard boxes to organics industry | | | Network developing; no extension services current; local markets, natural resources | | | Family employment, with both seasonal and full time employees | | | 90**%** | | |  |
| 5. | | | (H 57 Off) 40s + yrs, male and female, two children  Ed: second level | | | 500 acres; tillage; grazing | Five; cattle, sheep, miscanthus, cattle breeding, cash crops | | | Established network, Teagasc, LEADER, natural resources, productivity/processing in grain. | | | On and off-farm employment.  Excellent farm management. | | | **100%** | | |  |
| 6. | | | (H 63 Off) 60 yrs; malr & female; one child male 38 yrs +. Ed: third level. | | | 1,100 acres; tillage; forestry | Six; tillage, grazing, forestry, grain processing, hemp, miscanthus. | | | Extensive networking; local, national, international. Teagasc – natural resources. | | | On and off-farm employment.  Excellent management, planning, new ideas. Curious. | | | **100%** | | |  |
| 7. | | | (H101 May) 30 yrs +; male and female. Children three below 17 yrs. Ed: second Level. | | | 185 acres; grazing, tillage. | Three: dairy cattle, niche market in grain, suckler cattle. | | | LEADER, Teagasc. Developing network, primarily local; natural resources development. | | | On-farm employment.  Strong leadership and management skills. Determined. | | | 86**%** | | |  |
| 8. | | | (H 107 Off) 30 yrs +  Male & female  One child 17 yrs +  Ed: second level | | | 185 acres  grazing | Three  Dairy cattle, beef cattle, manufacturing business | | | Teagasc,  Developing network,  Natural wood resources | | | On and off-farm employment.  Welcome new challenge in manufacturing steel business. Known markets and customers. | | | **87%** | | |  |
|  |  |  |  | | |  |  |  |  |  |  |  |  |  |  |  |  |  |  |
| 9. | | | (H 118 Off) 40 yrs +  Male & female  One child 17 + years  Ed: third level | | | 130 acres  grazing | Three  Self-catering cottages, cleaning business, tourism marketing | | | LEADER, Failte Ireland, established national marketing network, natural and cultural resources | | | On-farm employment.  Successful businesses.  Ambitious. Energetic. | | | 86**%** | | |  |
| 10. | | | (H 103 Off) 40 yrs +  Two children 17 yrs +, Ed: third level | | | 87 acres  grazing | Four  Sheep, & cattle, B&B, Angling | | | LEADER, Failte Ireland, Fisheries Board, established marketing/distribution network, natural & cultural resources | | | On-farm employment. Well thought out product offering. Social. Enjoy challenge of new businesses. | | | 75**%** | | |  |
| 11. | | | (H 70 May)  Male & female, 30 + yrs,  Two children below 17 yrs, Ed: third level | | | 76 acres  grazing | Five,  B&B, Angling, forestry, special needs arts/crafts, special needs fishing | | | LEADER, Forestry Service, Fisheries Board, natural and cultural resources | | | On-farm employment.  Creative. Interested. Ambitious. Good planner and manager. | | | 89**%** | | |  |
| **Potential Innovative Diversifiers = 11 Households** | | | | | | | | | | | | | | | | | | |  |
| 12. | | (H 21 May) 40 yrs. Male & Female, one child male, 17 yrs +. Ed: third level. | | 75 acres | | | Sheep, cattle | | | | Networking, while limited in benefit returns, evidence of good animal husbandry and management. Enterprise Board, Forestry Service. | | Underemployment. No long-term planning. Technical management of enterprises inefficient. Acknowledges need to improve. | | | 65**%** | | |  |
| 13. | | (H 123 May) 40 yrs, Male & female. Two children under 17 yrs. Ed: second level. | | 50 acres.  Grazing, forestry. | | | Forestry, sheep, cattle. | | | | Networking, non-application of natural/cultural resources yet. However, good engagement with Forestry Service, Teagasc in exploring options. | | No long-term planning.  Evidence of good ability present, though. | | | 62**%** | | |  |
| 14. | | (H 67 Off) 30-40yrs, children under 17 yrs. Ed: second level | | 140 acres, grazing | | | Sheep, mixed crops, B&B | | | | Networking, low management ability of resources. While no business links with planning natural/cultural resource development/exploitation, evidence of identification of changes required. | | No long-term planning yet, but seems to be considering new options. | | | 57**%** | | |  |
| 15. | | (H 125 Off) 50 yrs +. Male & female, no children. Ed: second level. | | 78 acres, cultivation, forestry | | | Forestry, fodder crops, landscape conservation/protection, composting, animal breeding | | | | Networking, business future unsure, LEADER. In exploratory phase. | | No long-term business potential yet, but searching for new business. | | | 57**%** | | |  |
| 16. | | (H 108 May) 30 yrs +. Male & female, three children, below 17 yrs. Ed: second level. | | 260 acres, grazing, forestry | | | Sheep, cattle, forestry, mobile sawmill, hunting facility, accommodation | | | | Networking, LEADER, Teagasc, Forestry Service, no new initiatives or effective business structure, imitation over innovation | | Unclear as to future planning, loose management, under exploited natural/cultural resources. However, the farmer has unexploited potential to advance. Inward looking, but potential obvious. | | | 60**%** | | |  |
| 17. | | (H 4 Off) 30 yrs, male and female. Two children,,,,, under 17yrs. Ed: second level | | 300 acres,  Forestry, grazing, cultivation | | | Cattle, forestry, herbs, food processing. | | | | Developing network. LEADER, Teagasc, Forestry Service. Developing natural/cultural resources | | Future plans to develop food business, nothing current. | | | 45**%** | | |  |
| 18. | | (H 69 Off). Male & female 40 yrs +. Two children under 17 trs. Ed: second level. | | 22 acres, crops mixed, grazing | | | Horses, organic, self-catering cottages | | | | Low networking, local market access only. High agency dependency. Failte Ireland, LLEADER. | | Underemployed. However, evidence of ability to manage risk and deal with change. | | | 51% | | |  |
| 19. | (H 13 May) 40 yrs +. Male & female. One child over 17 yrs. Ed: second level. | | 73 acres.  Grazing | | | | | Sheep, cattle | | No evidence of extension service supports. Network of sorts established, but range limited. Unexploited natural/cultural resources. | | | | No evidence of future business planning at this stage. Does seem to be considering future options. Farm appears well run with potential identified beyond sheep and cattle by farmer. | | | 53**%** | | |
| 20. | (H 106 Off) 40 yrs +. Two children 17 yrs +. Ed: second level. | | 46 acres. Grazing, horticulture | | | | | Vegetables, cattle, self-catering wedding business. | | Networking potential., reconsidering business strategy, LEADER, Teagasc | | | | Potential to develop business in future marketing wedding business limited but developing.  Natural/cultural resources under developed acknowledged by household. | | | 49**%** | | |

| 21. (H 14 May) 60 yrs +. Female (widowed). One daughter 20s. Married, one child under 17 yrs. Ed: second level. Daughter third level. | 45 acres,  Grazing, cultivation | Horticulture, cattle, sheep, garden centre. | Teagasc, LEADER, natural/cultural resources, limited business development. | Daughter main manager. Potential for business development. Plans not well formed, but considering options. | **50%** |
| --- | --- | --- | --- | --- | --- |
| 22. (H 54 Off). 40yrs +. Male. No children. ED: third level. | 260 acres | Rollout lawn/grass. On-farm sales, sheep, cattle. | LEADER. Limited networking. | Employed on-farm. Was hit by recession. Potential to rejuvenate a good business evident. Planning future. | 60**%** |
| 23. (H 66 Off) 40 yrs +. Male & female. One child 17yrs +.  Ed: second level | 250 acres. Tillage, grazing. | Co-operative, dairy production, grain, beef cattle. | Poor networking.  Dependent on farm advisors.  Teagasc | On-farm employment. Better marketing required. Deciding to farm cooperatively or try going alone. Has networking ability, but under exploited at time of interview. | **55%** |

**Non-Innovative Diversifiers = 14 Households**

| 24. | (H 48 Off) 40 yrs +. Male & female. One child over 17 yrs. Ed: second level. | | 100 acres.  Grazing | | | Cattle.  Bed & Breakfast. | | Poor management & networking. | | Underemployed. Poor marketing | | 30**%** | |
| --- | --- | --- | --- | --- | --- | --- | --- | --- | --- | --- | --- | --- | --- |
| 25. | (H 178 Off) 40 yrs +. One child over 17 yrs. Ed: third level. | | Grazing | | | Suckler herd, dairying, self-catering accommodation | | Poor networking & management structure. Teagasc, Failte Ireland. | | On-farm employment. Better marketing required. Better management required. | | 30% | |
| 26. | (H 104 May) 45yrs +.  Two children 17 yrs. Ed: second level | | 80 acres | | | Cattle, sheep | | Poor management | | underemployed | | 28**%** | |
| 27. | | (H 71 Off) Male & female 50 yrs +. One child over 35 yrs. Ed: second level. | | 229 acres, tillage, horticulture | Three, cash crops, vegetables, cereal production | | No networking, future of market unsure. No strategy to address fall in market demand for produce. High levels of dependency on farm advisors for advice in managing farm. | | Under employed | | 24**%** | |  |
| 28. | | (H 115 May)  Male & female 40 + yrs  One child under 17 yrs, Ed: second level | | Land area non-specific,  Grazing, tillage | Cattle, cash crops | | Teagasc, no networking, limited social network, no business or business history | | Underemployed | | 1**5%** | |  |
| 29. | | (H 15 May)  Male & female 40 + yrs, one child below 17 yrs. Ed: second level. | | 116 acres,  grazing | sheep | | Teagasc | | On-farm employment  No initiative for change evident. | | **5%** | |  |
| 30. | | (H 16 May)  Female 60s + yrs, no children, Ed: not disclosed | | 305 acres,  grazing | Sheep | | Teagasc | | Land rented. Resigned to gain income from land rental. | | **5%** | |  |
| 31. | | (H 20 May) Male & female 50s, two children, age non-specific. Ed: first level | | 150 acres,  Forestry, grazing, tillage | Forestry, sheep, cash crops | | Forestry service, no networking | | Underemployed. Poor outlook. Abundant farm resources underdeveloped. | | 9**%** | |  |
| 32. | | (H 41 May) Male & female 40s. Three children under 17 yrs. Ed: second level. | | Acreage undisclosed. Grazing | Sheep | | No networking, no agency involvement disclosed | | On-farm employed | | 5**%** | |  |
| 33. | | (H 23 May) Male & female 40 + yrs. One child over 17 yrs. Ed: First level. | | 140 acres.  Grazing | Sheep,  cattle | | No networking, farm advisors | | Employed on- and off-farm.  No commitment shown for change. | | 5**%** | |  |
| 34. | | (H 33 May) Male 60s +. No children. | | 300 acres | Forestry | | No networking | | Semi-retired | | 5% | |  |
| 35.  36. | | (H 117 May) Male & female 30s + yrs.  Three children under 17 yrs.  (H 105 May)  Male & female  40s.  No children. | | Land area not specified  48 Acres | Sheep, cattle  Sheep, cattle | | No networking  Some Networking  Marketing marginal.  Confident. | | Employed on-farm. Community of similar farms. No evidence of individual thinking.  Requires complete rethink of  farming objectives. | | 8%  32%. | |  |
| 37. | | ( H 109 Off) Male & female 40 yrs +. Three children under 17 yrs. Ed: second level. | | 22 acres, grazing. | Sheep, cattle | | No networking | | Under employed. No evidence of anything new, planned or possible – requires rethink on networking, getting advice. | | 10**%** | |  |
