# Supplementary material for: A description of data sets to determine the innovative diversification capacity of farm households
Source: Data Brief. 2016 Jul 9;8:1088–93. doi: 10.1016/j.dib.2016.07.007 (PMC4970492; doi:10.1016/j.dib.2016.07.007)
Supplement: Supplementary file 2 — Supplementary material [file mmc2.zip › Zip File JRS June 27/Table 2 - Agency Interviews.docx]

**Table 2 –** The original agency/extension service interview structure showing the analytic procedure leading from the main topics to the specific research query

| Main topics | Subtopics | | Questions |
| --- | --- | --- | --- |
| The linkages between agencies/agencies/  Policy actors and farm household innovation | | Effectiveness of Irish innovation policy  Implementation of EU innovation policy. | How effective is your agency’s strategy in engendering/fostering/supporting innovation? What are the policy options? What is the role and function of your agency? What are its values (environmental, economic, technological, policy-led)? How workable is your strategy and how is it implemented? |
| Irish innovation strategy in implementation or interpretation of EU innovation policy? | | Innovation policy type  Policy delivery | How much inputs have you into your agency’s policy? How well is innovation understood at executive, middle and ‘grassroots’ levels? Define the main goals and objectives of your innovation strategy. How effective is it. What are your personal views on the RDR 1999, current CAP reforms, Agenda 2000? Are these views shared by other policy actors extra or intra agency?  Is policy always compatible with the aims/objectives and capacity for innovation equally between households? What are the differences and how effective is your agency in responding/dealing with cultural/social diversity?  How compatible is national innovation policy with EU innovation policy? Have you been briefed on both? How do you understand/conceptualise innovation and your agency’s role/function in it? How much input have you? |
| Appraisal of decision-making performance | | Input and involvement in decision-making | How effective is your agency in delivering on its policy objectives? Can you describe your involvement, its capacity to influence decisions, promote particular innovation types? |
| Appraisal of policy effectiveness | | Performance assessed | How do you assess households’ innovation performance? How is agency performance assessed? Are you assed (if yes) and by whom? Is there a need for further assessment? How might this be realised? |
| Policy evolution | | Uniformity of innovation policy | How does policy facilitate innovation sectorally? Is it sector specific? Has it changed/evolved much recently/historically? |
| State policy facilitation assessment | | State’s role in policy delivery | How effective is Ireland’s state structure to innovation? What does sustainability/resilience mean in State/EU innovation policy context? |
| Policy recommendations | | Recommendations at State and EU level. | How might policy need to be evolved? What recommendations do you suggest? |
