# Supplementary material for: A description of data sets to determine the innovative diversification capacity of farm households
Source: Data Brief. 2016 Jul 9;8:1088–93. doi: 10.1016/j.dib.2016.07.007 (PMC4970492; doi:10.1016/j.dib.2016.07.007)
Supplement: Supplementary file 2 — Supplementary material [file mmc2.zip › Zip File JRS June 27/Table 3 - Issues.docx]

| **Table 3 - ‘Category Index’ -Summary of the policy issues found affecting the appraisal of decision processes in relation to farm households’ capacity for innovation.** | | | | |
| --- | --- | --- | --- | --- |
| **Categories** | | **Subcategories dealing with agency/extension service innovation capacity based on responses** | **Results on Innovation** | |
| Lack of sufficient performance results | Inadequate/Denial | | | Negative |
| Insufficient data on innovation in different socio-economic and cultural contexts | Widespread | | | Limiting Effect Denial Common |
| Lack of consideration for reasons behind inaction | Common | | | Impacts on Diversity |
| Lack of agency performance assessment criteria | Inadequate | | | Makes Remedial Work more Difficult |
| Lack of agency performance measurement | Inadequate | | | Supports Vagueness indicating protectivism |
| Deficit in consideration of field research evidence | Widespread Denial | | | Limits understanding & support capacity |
| Lack of transparency and public accountability concern | Widespread Denial | | | Reaffirms dominant policy position |
| Lack of transparency in the administration of supports | Inadequate Denial | | | Limits understanding & support capacity |
| Rejection of alternative ideas and empirical research data | Widespread | | | Limiting effect |
| Lack of assessment of public/private partnership effects | Widespread | | | Perpetuates Status Quo/lack of reform |
| Lack of cohesive leadership | Inadequate Denial Common | | | All the above |
| Uncritical acceptance of European innovation model | Widespread | | | Limits Diversity (socio-economic/cultural) |
| Vague understanding of innovation | Inadequate | | | Negative effect |
| Reliance on dominant business models | Widespread | | | Negative effect |
| Priority political over socio-economic | Widespread | | | Negative effect |
| Policy adherence prioritised | Inadequate | | | Dependency on policy actors maintained |
| Lack of efficiency in administration/decision making | Widespread Denial Common | | | Negative effect |
| Policy conflicting with householder values | Inadequate Limited Acknowledgment | | | Negative to risk taking and trust in state |
| Erosion of trust in State not acknowledged | Common | | | Reaffirms negative effect |
| Over prioritization of techno-economic and big business innovation | Widespread Denial combined Contradictory | | | Negative effect on those less well resourced |
| Social innovation not considered | Widespread | | | Negative to networking & innovation diversity |
| Marginalization of diverse business possibilities and actions | Common | | | Negative to networking & innovation diversity |
| Short-term prescriptions over long-term | Common Denial | | | Negative effect |
| Staffing of management agencies narrow and political over diverse expertise value | Common Non-responsive | | | Supportive of already better resourced actors |
| Prioritization of agency interests | Widespread Denial | | | Negative to diversity of business forma and innovation |
| Lack of cohesive agency regulation and accountability | Common/Widespread Admitted | | | Leach out effect with negative/widespread rsults |
| Break in communication with business actors | Most Common at Executive Levels | | | Negative effect |
| Lack of acknowledgment of business actor position | Common at Executive Levels | | | Negative effect |
| Lack of performance feedback | The Norm | | | Negative effect – confusion resulting |
| Subjective appraisal of business successes | Widespread | | | Selective Support and Exclusive |
| Failures ignored | Common | | | Negative effect and innovation unrecognised |
| Lack of mechanism in determining successes and failures | Widespread | | | Negative effect and innovation unrecognised –questionable decision-making |
