# Supplementary material for: A description of data sets to determine the innovative diversification capacity of farm households
Source: Data Brief. 2016 Jul 9;8:1088–93. doi: 10.1016/j.dib.2016.07.007 (PMC4970492; doi:10.1016/j.dib.2016.07.007)
Supplement: Supplementary file 2 — Supplementary material [file mmc2.zip › Zip File JRS June 27/Table 4 - Farm Household Interview Schedule.doc]

Table 4 - The farm-household interview schedule showing the analytic procedure from the main topic to specific questions.

Main topics	Subtopics	Question Contexts

| Farm household characteristics in relation to diversification. | Household profile:  Age, member numbers, education, farm and non-farm work experience. | How did you reach the decision? What were your decision-making processes – motivations (e.g. household succession, market or policy or peer pressures, lifestyle choice), influences, management style, resources applied, organisational involvement, and business choice(s)? Can you tell me the farm & household’s history (family farm, how long in the family etc)? |
| --- | --- | --- |
| Transition from diversification to innovation. | Innovation characteristics.  Innovator characteristics. | How did you develop from this diversification to what it is now?  What plans have you in the short, long and medium terms for this new/novel business, or management strategy, or process? What are the relationships of it to sustainability, resource applications, skills sets present or being acquired, the farm household? What are your markets and where are they (local, regional, international)? Have you further plans for similar ‘enterprises’? What are they? How are you planning to implement them and why? |
| Environmental sustainability as a farm, household and decision issue. | Nature of the diversification/innovation – marketing, planning, its organisation. | How are you marketing it? How did you identify the market? Describe the business – seasonal, annual etc? How did you select it? Why this ‘diversification’? |
| Agency involvement and/or informal networking and other contacts. | Incentives, agency supports, advice, contacts, formal and informal contacts with peers or experts. Networking, actors involved, main sources of knowledge and information. | Have you been in contact with any development agency or extension service in relation to your decision? How many times, and how many agencies? Have they been influential, helpful or unhelpful? How much do they control your diversification/innovation? To what extent did others influence your thinking on the environment, business/household/farm succession, and who and what were the main influences on your decision making? |
| Overview of the processes described in relation to farming in the locality and region, the influence of market and policy forces. | Characteristics of the farm and its resources in local and regional context. Identification of unique social, economic, cultural and physical resources. | How have changes in policy influenced the development of resources by you on this farm? How have market forces defined your strategies? Is conservation an issue and in what context? Has CAP policy and its reforms favoured your type of farming? What changes have you experienced? What is the future of your farm? Are you planning to expand or develop into non-agricultural business activities? Is there a future for your children o this farm? Is this a concern for you? At what age should a farm be handed on to the next generation? What are the advantages or disadvantages between young and older farmers? What role does each member of the household have in the daily running of this farm? What divisions of labour are there and how much work is contracted? What decision capacity has your wife or husband? What type of employment has been created by these enterprises? |
