# Supplementary material for: A description of data sets to determine the innovative diversification capacity of farm households
Source: Data Brief. 2016 Jul 9;8:1088–93. doi: 10.1016/j.dib.2016.07.007 (PMC4970492; doi:10.1016/j.dib.2016.07.007)
Supplement: Supplementary file 2 — Supplementary material [file mmc2.zip › Zip File JRS June 27/Table 5 - Overview of characteristics of survey participants.doc]

**Table 5 - Overview of characteristics of postal survey respondents and interview participants**

_____________________________________________________________________________________________________________________________________________________________________________

Mayo	Offaly__________________________

Total number of Diversifiers from the 37: 15	11

Total number of Innovative Diversifiers from the 37: 3	8

Number and percentage of postal respondents by county:

Number: Percentage: Sent: 200	100

Number: Percentage: Returned: 109 (54.5%)	50 (50%)

Number and percentage of interview respondents by county from 36: _____________________18 (50%)____________ 18 (50%)________________________

Number of non- agricultural and agricultural activity types from 1) the 11 interview respondents overall: 2) the diversifiers

Non-agricultural (Heritage/Tourism/Activity): (ID) 8	(ID) 5

Agricultural (Grazing/Tillage/Organics): (ID) 7	(ID) 10

Environmental (Forestry/Direct/Marketing/Conservation):________________ ___________ (ID) 5_______________________ __(ID) 7__________________________

Percentage of interview respondents by one member fully occupied:

Full-time: (ID) 2	(ID) 4

Part-time: (ID) 1	(ID) 4

Average farm size: (ID) 172 Acres	(ID) 333.75 Acres

___________________________________________________________________________________________________________________________________________

(ID) 45	(ID) 46

Average Age:_________________________________________________________________________________________________________________________________

Second Level	Third Level+	Second Level	Third Level +

Education:___________________________	(ID) 1_(D) 10 (1 below 2^nd^ level; 1 non res)_	__	(ID) 2_(D) 4_________(ID) 3 (D) 8 (2 non res)_______(ID) 5 (D) 3

Average number of agencies involved per-household:____________________________________(ID) 2.75 (D) 1.25________________(ID) 1.4 (D) .90_________________

Average number of children:________________________________________________________(ID) 3 (D) 1.5____________________(ID) 1.5_(D) 1.3__________________

Average age of children on-farm:____________________________________________________(ID) 33_(D)_19.25_________________(ID) 20_(D) 17__________________
